# Book Review: How Men Age: What Evolution Reveals about Male Health and Mortality

**DOI:** 10.3389/fpsyg.2017.00894

**Published:** 2017-05-30

**Authors:** Farid Pazhoohi, Joana Arantes

**Affiliations:** Human Cognition, School of Psychology, University of MinhoBraga, Portugal

**Keywords:** men aging, male health and mortality, reproductive senescence, biological anthropology, Darwinian evolutionary theory, life history theory

Nothing would be more interesting than reading a book on men aging by an author who is an expert on comparative male life histories. Richard G. Bribiescas is a Professor of Anthropology, Ecology and Evolutionary Biology at Yale University, and has conducted research in evolutionary biology and endocrinology of human, as well as comparative studies on reproduction, growth, aging, and metabolism for many years. He is well-known for his research on male aging and reproductive senescence.

In the first chapter, Bribiescas explains what this book is all about and why Darwinian evolutionary theory is needed to gain a deeper understanding of male health, illness and aging. Additionally, he explains why it is important to consider aging across species and cross-culturally. While Bribiescas briefly explains how natural selection works, defines what he means by aging and concepts such as aging, life history theory and adaptation, he also lists the contents of the book by highlighting the points that he is going to extend in the upcoming chapters.

By implanting the seed of curiosity in the reader's mind during the first chapter, Bribiescas begins the second chapter by explaining why aging happens from a biological perspective. He explains why aging is pertinent to sexually reproducing organisms and how sexual reproduction and energetic investment in reproductive effort affect the life span, rate of aging and mortality. Here, Bribiescas considers aging as the “decline in physiological function that occurs with the passage of time” (p.22), rather than the calendrical passage of time, which is dependent on organisms' metabolic rate and body size.

Then he turns to male aging and by providing evidence from modern populations, hunter-gatherer populations, primates, and the archeological records for higher male mortality—compared with that of female—argues why men and women do not have the same life spans, and why men have a higher tendency to expose themselves to risky endeavors than women.

In the third chapter, Bribiescas deals with the physiological changes that occur as men age, such as increase of fat tissues, muscle loss and hormonal change and explains how decreased hormonal plasticity is responsible for less efficient metabolism as they get older. Accordingly, he discusses why and how men-specific metabolic rates contribute to higher mortality in men.

Bribiescas reasons that investment in sexually dimorphic trait is adaptive for men which in return needs higher metabolic rates and higher oxidative stress compared to women, hence shorter life spans and higher mortality rates. Then he masterfully and concisely explains how aging is associated with muscle mass loss, fat deposition and testosterone decline.

In the next chapter Bribiescas investigates the evolutionary advantages of living longer lives to our species. Also, he explains how older men contributed to evolution of long life span in humans and relates long life spans to men's ability in reproducing at older ages.

In the fifth chapter, the author considers the role of older men in the evolution of paternal investment. After explaining the evolution of pair bonding and paternal investment, he proposes the role of men aging in evolution of pair bonding and paternal care. Here he suggests that fat deposition associated with aging is responsible for physiological and behavioral changes toward paternal care and investment. Bribiescas argues that this hormonal change in men associated with aging is to support male longevity.

In the sixth chapter, Bribiescas considers the health issues of men aging from an evolutionary point of view. He reviews the biological and physiological aspects of men shorter aging and, by considering the effect of testosterone on men bodies, argues how men compared to women are also poorer in terms of survivability. Moreover, in this chapter he discusses different aspects of older men's health and wellbeing.

In the last and concluding chapter Bribiescas discusses the important role of older men in shaping human evolutionary future. Specifically, he highlights the important role of elderly in controlling resources and conflicts in shaping human societies.

Bribiescas is a great storyteller and masterfully puts all of his explanations into stories of his own and others' lives. This book is full of amazing hindsight about aging and healthy aging backed with scientific facts. All the arguments throughout the book are supported by laboratory, cross-cultural, and longitudinal findings. Although the three last chapters lack take-home concluding messages, after reading this book you would learn much about men body functions and aging, and the contributing factors.

This interesting book is an endeavor to familiarize the reader with the physiological and underlying causes of men aging while at the same time avoiding readers fearing of the technical scientific terms and concepts. However, the potential audience of the book are educated readers, being highly recommended to biological anthropologists, evolutionary biologists and evolutionary psychologists.

## Author contributions

FP provided the first draft and JA revised the manuscript. Both authors approved the final version.

### Conflict of interest statement

The authors declare that the research was conducted in the absence of any commercial or financial relationships that could be construed as a potential conflict of interest.

